# The vulnerability of men to virologic failure during antiretroviral therapy in a public routine clinic in Burkina Faso

**DOI:** 10.7448/IAS.17.1.18646

**Published:** 2014-01-15

**Authors:** Pauline Penot, Arsène Héma, Guillaume Bado, Firmin Kaboré, Ibrahim Soré, Diamasso Sombié, Jean-Richard Traoré, Jean-Baptiste Guiard-Schmid, Arnaud Fontanet, Laurence Slama, Adrien Bruno Sawadogo, Christian Laurent

**Affiliations:** 1 Hôpital de jour du service de médecine interne du CHU Sanou Souro, Bobo-Dioulasso, Burkina Faso;; 2 UMI 233, Institut de Recherche pour le Développement (IRD), Université Montpellier 1, Montpellier, France; 3 Initiatives Conseil International – Santé, Ouagadougou, Burkina Faso; 4 Emerging Diseases Epidemiology Unit, Institut Pasteur, Paris, France; 5 Conservatoire National des Arts et Métiers, Paris, France; 6 Service des maladies infectieuses et tropicales, hôpital Tenon, Paris, France

**Keywords:** HIV, antiretroviral, efficacy, virologic failure, gender, Africa

## Abstract

**Introduction:**

Gender differences in antiretroviral therapy (ART) outcomes are critical in sub-Saharan Africa. We assessed the association between gender and virologic failure among adult patients treated in a public routine clinic (one of the largest in West Africa) in Burkina Faso.

**Methods:**

We performed a case-control study between July and October 2012 among patients who had received ART at the Bobo Dioulasso Day Care Unit. Patients were eligible if they were 15 years or older, positive for HIV-1 or HIV-1+2, and on first-line ART for at least six months. Cases were all patients with two consecutive HIV loads >1000 copies/mL (Biocentric Generic or Abbott Real Time assays), or one HIV load >1000 copies/mL associated with immunologic or clinical failure criteria. Controls were all patients who only had HIV loads <300 copies/mL. The association between gender and virologic failure was assessed using a multivariate logistic regression, adjusted on age, level of education, baseline CD4+ T cell count, first and current antiretroviral regimens and time on ART.

**Results:**

Of 2303 patients (74.2% women; median age: 40 years; median time on ART: 34 months), 172 had virologic failure and 2131 had virologic success. Among the former, 130 (75.6%) had confirmed virologic failure, 38 (22.1%) had viro-immunologic failure, and four (2.3%) had viro-clinical failure. The proportion of men was significantly higher among the cases than among the controls (37.2% vs. 24.9%; *p<*0.001). Compared to controls, cases were also younger, more immunodeficient at ART initiation, less likely to receive a protease inhibitor-based antiretroviral regimen and had spent a longer period of time on ART. After adjustment, male gender remained strongly associated with virologic failure (odds ratio 2.52, 95% CI: 1.77–3.60; *p<*0.001).

**Conclusions:**

Men on ART appeared more vulnerable to virologic failure than women. Additional studies are needed to confirm the poorer prognosis of men in this setting and to determine the causes for their vulnerability in order to optimize HIV care. From now on, efforts should be made to support the adherence of men to ART in the African setting.

## Introduction

Gender differences in antiretroviral therapy (ART) are increasingly recognized as a critical issue in sub-Saharan Africa, where 7.5 million HIV-positive patients are now being treated [1,2]. In this region, men are more disadvantaged in access to treatment compared to women (although the latter face social inequalities). By the end of 2011, men comprised only 36% of the patients receiving ART but accounted for 44% of those eligible for treatment [2]. Men also tend to start ART with more advanced stages of HIV disease than women [3,4], partly because they are less frequently tested for HIV [5].

Moreover, there is a growing body of evidence to suggest that men who access ART in sub-Saharan Africa are more at risk of therapeutic failure than women, independently of their later initiation of treatment. They are more likely to interrupt ART, to be lost to follow-up, to have poorer immunologic responses and are more likely to die than women [6–21]. However, a recent study in South Africa suggested that the higher male mortality rate on ART might be related to background differences in death rates between men and women in this context rather than to lower ART efficacy in men [11].

Evidences of gender differences in virologic outcomes on ART in sub-Saharan Africa are limited and conflicting, with studies showing no gender difference and others showing poorer virologic outcomes among men than among women [11,13,22–29]. Additional data are needed to inform clinical outcomes and adapt patient management to improve the quality of care and the effectiveness of treatment programmes. We therefore assessed the association between gender and virologic failure in adult patients receiving ART in a public routine clinic in Burkina Faso.

## Methods

### Study design

A case-control study was performed between July and October 2012 among patients who had received ART at the Day Care Unit of the Sanou Souro University Hospital in Bobo Dioulasso (the second largest city in Burkina Faso). Routine prescription of ART started in 2002 (few patients were treated between 1999 and 2002), and this clinic now has one of the largest West African cohorts of patients and serves as a reference HIV clinic. HIV load measurements started in July 2008 in the context of an active screening of patients with suspected therapeutic failure. From September 2010, HIV load measurements were progressively scheduled once a year for all the patients on ART (starting with those who did not have measurements in the previous period) but this recommendation was applied at the physician's discretion and subject to the availability of tests. Patients were eligible for the study if they were 15 years or older, positive for HIV-1 or HIV-1+2, in care between July 2008 and June 2012, and on first-line ART for at least six months.

Cases were all the patients who had either two consecutive HIV loads >1000 copies/mL, one to six months apart (*confirmed virologic failure*), one HIV load >1000 copies/mL associated with a WHO-defined immunologic failure criterion, three months apart (*viro-immunologic failure*), or one HIV load >1000 copies/mL associated with a clinical failure criterion (i.e. a WHO clinical stage 4 adverse event, pulmonary tuberculosis, severe bacterial infection or death), three months apart (*viro-clinical failure*), at any time point after six months on treatment [30]. Priority was given to cases of confirmed virologic failure followed by cases of viro-immunologic failure and viro-clinical failure. Controls were all the patients who only had HIV loads <300 copies/mL (limit of detection of the Biocentric assay). Patients who had HIV loads between 300 and 1000 copies/mL, those who had isolated HIV loads >1000 copies/mL without concomitant immunologic or clinical failure and those for whom HIV loads >1000 copies/mL were followed up by HIV loads <1000 copies/mL without ART switch were excluded.

### Procedures

Patients attended clinical visits at ART initiation, between two weeks and four months after initiation according to the patient's health conditions and then twice a year. Patients could also attend the clinic at any time that they felt unwell. HIV load was measured using a generic HIV-1 viral load assay (Biocentric, Bandol, France) or the Abbott RealTime HIV-1 quantitative assay (Abbott Molecular, Des Plaines, IL, USA; limit of detection, 40 copies/mL). CD4+ T cell count were measured using a FACSCount device (Becton Dickinson, San Jose, CA, USA) or a CYFLOW counter (Partec, Münster, Germany) at ART initiation and every six months thereafter. The first-line antiretroviral regimens mostly included two nucleoside reverse transcriptase inhibitors (NRTIs) and one non-NRTI (NNRTI), but the latter drug was sometimes replaced by a protease inhibitor (PI) or a third NRTI. Adherence support was provided at ART initiation, two and four weeks later, and then on average every two months. Patients who did not attend scheduled appointments for drug supply were telephoned or visited at home. ART, biologic exams and other care were provided free of charge. Demographic and medical data were recorded in both individual paper and electronic (ESOPE, Epiconcept, France) medical charts.

### Statistical analysis

Eligible patients were identified using the ESOPE database. Their data were then extracted and checked using paper medical charts and laboratory, pharmacy and psycho-social databases, as well as by interviewing the clinic staff members (physicians, nurses, biologists, pharmacists and community health workers).

Between-group comparisons of patient characteristics were performed using the Chi-squared or Fisher exact test for the categorical variables and the Mann–Whitney test for the continuous variables. A multivariate logistic regression model was then used to compare virologic failure between men and women, adjusted on the following variables: age (<35 vs. ≥35 years), level of education (lower than secondary school vs. secondary school or higher), baseline CD4+ T cell count (≤200 vs. >200 cells/µL), first and current antiretroviral regimens (NNRTI-based, PI-based or other) and time on ART. A backward elimination procedure was used to determine the final model containing only gender, together with significant covariates and potential confounders.

The time on ART was calculated as the number of months between ART initiation and the following events as appropriate: first of two consecutive HIV loads >1000 copies/mL or single HIV load >1000 copies/mL associated with an immunologic or a clinical failure criterion (for the patients with virologic failure); first HIV load <300 copies/mL (for the patients with virologic success); switch to a second-line regimen, transfer to another clinic, last clinical visit for the patients lost to follow-up, death or database cut-off date for this study (June 20, 2012) (for the patients eligible but not included). All analyses were performed with STATA^©^ 12.2 (StataCorp, College Station, TX, USA) software.

## Results

### Characteristics of the study population

**Figure 1:**
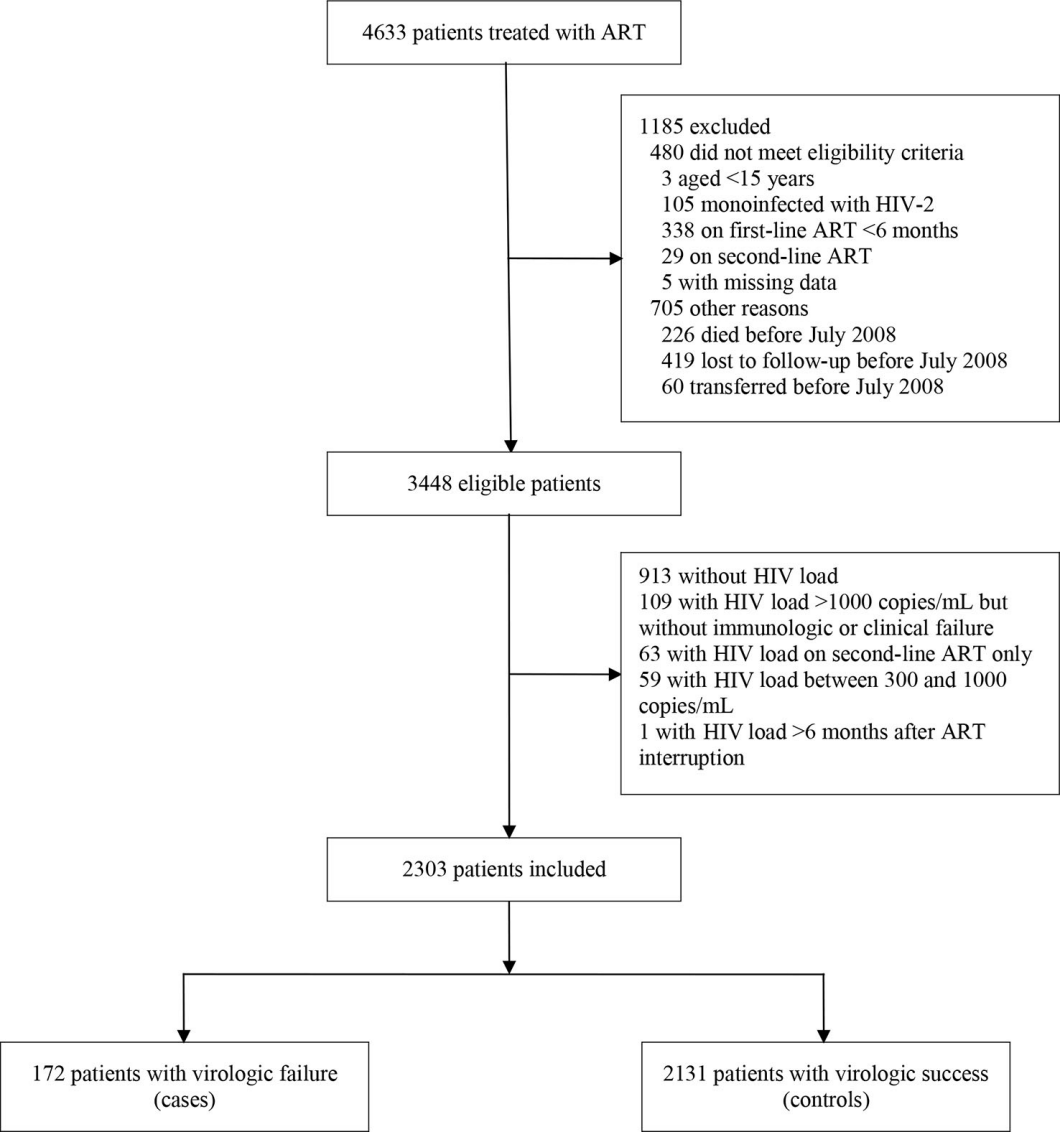
Study flow chart, Bobo Dioulasso Day Care Unit, Burkina Faso, 2008–2012.

Of 4633 patients who had received ART in the Bobo Dioulasso Day Care Unit, 3448 (74.4%) were eligible for the study (Figure 1). Of them, 2303 (66.8%) were included. These patients were more likely to be women (74.2% vs. 69.3%; *p=*0.003), slightly older (median age 40 years, interquartile range [IQR] 34–46 vs. 38 years, IQR 32–46; *p<*0.001), more likely to have reached at least the secondary school level (30.5% vs. 27.0%; *p=*0.032) and on ART for a longer duration (median time 34 months, IQR 15–55 vs. 18 months, IQR 8–37; *p<*0.001) than their counterparts who were not included.

There were 172 cases (patients with virologic failure) and 2131 controls (patients with virologic success). Among the cases, 130 (75.6%) had a confirmed virologic failure, 38 (22.1%) had a viro-immunologic failure and 4 (2.3%) had a viro-clinical failure. Only one case and 49 controls were dually positive for HIV-1 and HIV-2, while all the other patients were positive for HIV-1 alone.

The proportion of men was significantly higher among the cases than among the controls (37.2% vs. 24.9%; *p<*0.001; Table 1). Compared to controls, cases were also younger (median age 38 vs. 40 years; *p=*0.002), more immunodeficient at ART initiation (median CD4+ T cell count 126 vs. 149 cells/µL; *p=*0.021), more likely to have received a first NNRTI-based regimen (97.1% vs. 93.4%; *p=*0.034) and to receive a NNRTI-based regimen at the time of the study (96.5% vs. 91.8%; *p=*0.028) and had spent a longer time on ART (median time 42 vs. 33 months; *p=*0.001). By contrast, the level of education did not differ significantly between cases and controls (*p=*0.111).

**Table 1:** Patient characteristics by virologic status, Bobo Dioulasso Day Care Unit, Burkina Faso, 2008–2012

	Patients with virologic failure (*n=*172)	Patients with virologic success (*n=*2131)	*p*
Gender			
Women	108 (62.8%)	1601 (75.1%)	<0.001
Men	64 (37.2%)	530 (24.9%)	
Age (years)	38 (31–44)	40 (35–46)	0.002
<35	69 (40.1%)	541 (25.4%)	<0.001
≥35	103 (59.9%)	1590 (74.6%)	
Level of education^a^			
Less than secondary school	128 (74.8%)	1470 (69.0%)	0.111
Secondary school or higher	43 (25.1%)	660 (31.0%)
Baseline CD4+ T cell count (cells/µL)	126 (65–198)	149 (79–220)	0.021
≤50	28 (16.3%)	319 (15.0%)	0.010
51–200	91 (52.9%)	1062 (49.8%)	
201–350	35 (20.3%)	578 (27.1%)	
>350	1 (0.6%)	65 (3.0%)	
Unknown	17 (9.9%)	107 (5.0%)	
First antiretroviral regimen			
2 NRTIs+1 NNRTI	167 (97.1%)	1990 (93.4%)	0.034
2 NRTIs+1 PI	4 (2.3%)	136 (6.4%)	
Other	1 (0.6%)	5 (0.2%)	
Current antiretroviral regimen			
2 NRTIs+1 NNRTI	166 (96.5%)	1957 (91.8%)	0.028
2 NRTIs+1 PI	6 (3.5%)	174 (8.2%)	
Time on ART (months)	42 (23–59)	33 (15–55)	0.001

Data are *n* (%) or median (interquartile range).

a Data missing for one case and one control.

NRTIs, nucleoside reverse transcriptase inhibitors; NNRTI, non-nucleoside reverse transcriptase inhibitor; PI, protease inhibitor; ART, antiretroviral therapy.

Compared to women (Table 2), men were older (median age 44 vs. 38 years; *p<*0.001), more likely to have reached at least the secondary school level (38.9% vs. 27.6%; *p<*0.001) and more immunodeficient at ART initiation (median CD4+ T cell count 120 vs. 156 cells/µL; *p<*0.001). Conversely, no gender differences were observed in terms of first and current antiretroviral regimens (NNRTI-based regimen, 94.1% vs. 93.5%; *p=*0.486 and 92.9% vs. 91.9%; *p=*0.432, respectively) and time on ART (median 33 vs. 34 months; *p=*0.817).

**Table 2:** Patient characteristics by gender, Bobo Dioulasso Day Care Unit, Burkina Faso, 2008–2012

	Women (*n=*1709)	Men (*n=*594)	*p*
Age (years)	38 (33–44)	44 (39–50)	<0.001
<35	552 (32.3%)	58 (9.8%)	<0.001
≥35	1157 (67.7%)	536 (90.2%)	
Level of education^a^			
Less than secondary school	1236 (72.4%)	362 (61.1%)	<0.001
Secondary school or higher	472 (27.6%)	231 (38.9%)	
Baseline CD4+ T cell count (cells/µL)	156 (87–226)	120 (56–198)	<0.001
≤50	228 (13.3%)	119 (20.0%)	<0.001
51–200	857 (50.1%)	296 (49.8%)	
201–350	488 (28.6%)	125 (21.0%)	
>350	63 (3.7%)	3 (0.5%)	
Unknown	73 (4.3%)	51 (8.6%)	
First antiretroviral regimen			
2 NRTIs+1 NNRTI	1598 (93.5%)	559 (94.1%)	0.486
2 NRTIs+1 PI	105 (6.1%)	35 (5.9%)	
Other	6 (0.4%)	0	
Current antiretroviral regimen			
2 NRTIs+1 NNRTI	1571 (91.9%)	552 (92.9%)	0.432
2 NRTIs+1 PI	138 (8.1%)	42 (7.1%)	
Time on ART (months)	34 (15–55)	33 (15–56)	0.817

Data are *n* (%) or median (interquartile range).

a Data missing for one woman and one man.

NRTIs, nucleoside reverse transcriptase inhibitors; NNRTI, non-nucleoside reverse transcriptase inhibitor; PI, protease inhibitor; ART, antiretroviral therapy.

### Association between gender and virologic failure

By univariate logistic regression analysis, male gender was significantly associated with virologic failure (odds ratio [OR] 1.79, 95% confidence interval [95% CI] 1.29–2.48; *p<*0.001). Age, CD4+ T cell count at ART initiation, first and current antiretroviral regimens and time spent on ART were also associated with virologic failure in crude analysis while level of education was not (Table 3).

**Table 3:** Univariate and multivariate logistic regression analyses of factors associated with virologic failure in adult patients on ART, Bobo Dioulasso Day Care Unit, Burkina Faso, 2008–2012

	Univariate			Complete multivariate model^a^			Final multivariate model^b^		
			
	OR	95% CI	*p*	aOR	95% CI	*p*	aOR	95% CI	*p*
Gender									
Women	1.00			1.00			1.00		
Men	1.79	1.29–2.48	<0.001	2.43	1.70–3.48	<0.001	2.52	1.77–3.60	<0.001
Age (years)									
≥35	1.00			1.00			1.00		
<35	1.97	1.43–2.71	<0.001	3.10	2.17–4.44	<0.001	3.06	2.14–4.37	<0.001
Level of education									
Secondary school or higher	1.00			1.00			1.00		
Lower than secondary school	1.34	0.93–1.91	0.112	1.68	1.16–2.44	0.006	1.67	1.16–2.42	0.006
Baseline CD4+T cell count (cells/µL)									
>200	1.00			1.00					
≤200	1.51	1.03–2.21	0.034	1.38	0.93–2.07	0.112			
Unknown	2.79	1.52–5.13	0.001	1.78	0.90–3.54	0.097			
First antiretroviral regimen									
2 NRTIs+1 PI	1.00			1.00			1.00		
2 NRTIs+1 NNRTI	2.85	1.04–7.81	0.041	1.87	0.54–6.47	0.321	2.88	1.05–7.94	0.040
Other	6.80	0.64–72.46	0.112	5.56	0.47–65.18	0.172	6.50	0.58–72.30	0.128
Current antiretroviral regimen									
2 NRTIs+1 PI	1.00			1.00					
2 NRTIs+1 NNRTI	2.46	1.07–5.64	0.033	1.73	0.62–4.83	0.297			
Time on ART (per one-year increase)	1.12	1.04–1.20	0.002	1.15	1.06–1.24	0.001	1.18	1.09–1.27	<0.001

NRTIs, nucleoside reverse transcriptase inhibitors; NNRTI, non-nucleoside reverse transcriptase inhibitor; PI, protease inhibitor; ART, antiretroviral therapy.

a The association between gender and virologic failure was adjusted on all covariates.

b The association between gender and virologic failure was adjusted on significant covariates only.

Male gender remained strongly associated with virologic failure (OR 2.43, 95% CI: 1.70–3.48; *p<*0.001) after adjustment for age, level of education, CD4+ T cell count at ART initiation, first and current antiretroviral regimens and time spent on ART (Table 3). The result was quite similar in the final multivariate model (OR 2.52, 95% CI: 1.77–3.60; *p<*0.001) including age, level of education, first antiretroviral regimen and time spent on ART.

## Discussion

This study in adult patients receiving ART in a public routine clinic in Burkina Faso showed a higher risk of virologic failure among men than among women. The vulnerability of men was independent of age, level of education, CD4+ T cell count at ART initiation, first and current antiretroviral regimens and time spent on ART.

Our finding was consistent with other but not all African studies. In particular, very comparable strengths of association between gender and virologic response were reported in the DART (Development of AntiRetroviral Therapy in Africa) study in Uganda and Zimbabwe (adjusted OR 2.87, 95% CI: 1.66–4.98; *p<*0.001) and in another study in Uganda (crude OR 2.14, 95% CI: 0.99–4.63; *p=*0.05) [22,23]. Also, measurable HIV loads were found in 25.7% of men vs. 16.9% of women (*p=*0.012) in the Democratic Republic of Congo and in 55% of men vs. 31% of women (*p=*0.003) in Tanzania [24,25]. Conversely, gender was not associated with virologic failure in studies in South Africa, Malawi and Senegal [11,13,26,28,29]. It is worth noting that, to our knowledge, no studies have found a higher risk of virologic failure in women in this setting.

Two key elements might explain the vulnerability of men to virologic failure. The first element relates to gender differences in pharmacokinetic and pharmacodynamic profiles of antiretroviral drugs [31,32]. Higher concentrations of antiretroviral drugs which favour virologic efficacy [33,34] have been observed in women than in men. The second element relates to a worse adherence to HIV care and treatment in men, as frequently observed in Africa [6,35,36]. However, few studies have found either no difference between men and women or even a better adherence in the former [16,37]. Poorer male adherence to HIV care and treatment could be related to gender-based socio-cultural norms including the background reluctance of men to access healthcare and treatment, to a more difficult communication with health care workers (especially with female ones) on disease and treatment issues and to job constraints (e.g. working hours and lack of disclosure of HIV infection to the employer and colleagues) [38]. Unfortunately, we were not able to investigate the causes for the vulnerability of men to virologic failure in our study because measures of adherence and antiretroviral drug concentrations were not available.

Our study, based on a chart review in a routine-care setting, has several limitations. First, our results might not be fully generalizable. A quarter of eligible patients were excluded because they did not have HIV load measurements. Baseline characteristics of these patients were slightly different than those of included patients and, more importantly, their duration of ART was shorter (18 vs. 34 months). On the other hand, there might be different groups of patients because HIV load measurements were performed selectively, depending on the time periods. How this selection bias affected the association between gender and virologic failure is unclear. Second, biological or clinical events might have been under-reported. This information bias was minimized by analyzing all available paper and electronic files and by interviewing the clinic staff members. Third, because of the progressive strategy for HIV load measurements, we performed a case-control study that is less reliable than a prospective cohort study. However, we included all patients who fulfilled our definitions of virologic failure (cases) and virologic success (controls); took into account all available data for HIV load, CD4+ T cell count and clinical events; and adjusted the analyses for age, level of education, CD4+ T cell count at ART initiation, first and current antiretroviral regimens and time spent on ART. Finally, as already stated above, antiretroviral drug concentrations and adherence were not available; this prevented us from investigating the causes for the higher virologic failure in men than in women.

## Conclusions

Men on ART appeared more vulnerable to virologic failure than women, regardless of their HIV disease stage at ART initiation. Additional studies are needed to confirm the poorer prognosis of men in this setting and to determine the causes for their vulnerability in order to optimize HIV care. Efforts should be made to support the adherence of men to ART in the African setting.
